# Self-Objectification, Body Surveillance, and Body Shame Across Countries: A Comparison Between US, UK, Belgian, Israeli, and Thai Women

**DOI:** 10.5334/pb.1387

**Published:** 2026-03-04

**Authors:** Robin Wollast, Liesje Coertjens, Philippe Bernard, Anat Talmon, James J. Gross, Olivier Klein

**Affiliations:** 1 Stanford University, US; 2 Université libre de Bruxelles, BE; 3 Université catholique de Louvain, BE; 4 The Hebrew University of Jerusalem, IL

**Keywords:** Self-objectification, Body surveillance, Body shame, Cross-national study, Culture

## Abstract

Cross-national research on self-objectification remains notably limited. The present study investigated the associations between self-objectification, body surveillance, and body shame among women in Belgium (*N* = 239), the United Kingdom (*N* = 213), and the United States of America (*N* = 159) in Study 1, and in Belgium (*N* = 209), Israel (*N* = 299), and Thailand (*N* = 230) in Study 2. In Study 1, employing the Likert version of the Self-Objectification Questionnaire (LSOQ), we demonstrated that self-objectification indirectly predicts body shame through body surveillance in the case of Belgian, UK, and US women. In Study 2, we successfully replicated these indirect effects among Belgian, Israeli, and Thai women. This research stands as one of the first empirical, cross-national investigations of the improved self-objectification scale, evidencing the robust association between self-objectification, body surveillance, and body shame across countries.

According to objectification theory (Fredrickson & Roberts, 1997), Western women live in a society that emphasizes beauty and appearance (e.g., Bernard & Wollast, 2019) mainly through exposure to sexually objectifying media (Ward, 2016), but also within interpersonal interactions (Gervais et al., 2020; Riemer et al., 2022). These repeated instances of objectification frequently prompt women to internalize this perspective, viewing their bodies as objects subject to evaluation by others (Daniels et al., 2020; Szymanski et al., 2011). Engaging in self-objectification not only negatively impacts how women perceive themselves, but also lays the groundwork for detrimental mental health outcomes such as eating disorders and sexual dysfunction (Moradi & Huang, 2008). Since most studies in the field of self-objectification have been conducted among US women (for a review, see Roberts et al., 2018), the literature suffers from a lack of knowledge about this phenomenon among women from different national backgrounds. Based on two studies, the present work aims to fill this gap by examining the mediating role of body surveillance in the relation between self-objectification and body shame, in five nations, namely the United States of America (US), the United Kingdom (UK), Belgium, Israel, and Thailand.

## Self-Objectification, Body Surveillance, and Body Shame

Self-objectification involves adopting a third-person perspective on the self with a focus on one’s physical appearance rather than personal thoughts and feelings (Fredrickson & Roberts, 1997). This phenomenon is strongly associated with body surveillance, the most common behavioral manifestation of self-objectification, which can result in enduring consequences, notably body shame (see Moradi & Huang, 2008 for a review). More specifically, elevated levels of self-objectification manifest as heightened body surveillance, subsequently intensifying feelings of body shame (Wollast et al., 2021). McKinley and Hyde (1996) suggest that women engage in surveillance of their own bodies as if they were external observers; they think about how they look to others by comparing themselves with beauty standards and other women. Women high in body surveillance experience feelings of body shame when they realize that there is a disparity between these beauty standards and their perception of their own bodies. At this point, it is well-established that, in Western countries, self-objectification, body surveillance, and body shame are strongly associated (Wollast et al., 2021).

Despite a large body of research interest in self-objectification and its resulting consequences, the field suffers from a lack of cultural breadth as the majority of studies focusing on self-objectification have relied on Western populations (Loughnan et al., 2015; Moradi & Huang, 2008; Kahalon & Klein, 2025). Understanding how self-objectification operates in different national backgrounds would reinforce the cross-national generalizability of objectification theory and also point to improvements that could improve its applicability across national and cultural borders (Moradi & Huang, 2008).

## Self-Objectification across Countries

As American standards of beauty influence other cultures, researchers have investigated whether self-objectification is exclusive to Western societies. Loughnan et al. (2015) conducted a comparative study involving four Western countries (the US, the UK, Australia, Italy) and three non-Western countries (India, Japan, Pakistan), revealing that cultural factors strongly influence self-objectification, which seems predominantly prevalent in Western societies. Specifically, self-objectification levels were higher in the US and the UK as compared to other countries. Similarly, more recent cross-national research involving the US reported converging patterns of body surveillance and body shame across samples of undergraduate women from the United States, Belgium, Russia, and Thailand (Wollast et al., 2020b). In particular, women in the United States reported the highest levels of body surveillance and body shame, whereas Thai participants reported the lowest levels, suggesting that self-objectification is present across cultures, while tending to be reported at higher levels in American samples, where the instrumentalization of bodies through sexualization is particularly salient. Notably, Crawford et al. (2009) observed less body surveillance but *higher* body shame among Nepalese women than among US women.

In this context, a growing body of literature has demonstrated the prevalence of self-objectification, body surveillance, and body shame beyond the United States. For instance, research has documented the presence of body shame in Italy and Romania (Gattino et al., 2017). Similarly, Zheng and Sun (2017) applied objectification theory to investigate disordered eating and depressed mood among Chinese undergraduates, finding that body shame and appearance anxiety mediated the relationships between body surveillance, disordered eating, and depressed mood. Additionally, Teng and Poon (2017) found that body surveillance among young Chinese women predicted social anxiety through body shame and the need to belong (see also Li & Xiao, 2023; Xiao et al., 2023). Within this frame of reference, the current study enriches objectification theory by examining cross-national comparisons of self-objectification, body surveillance, and body shame in the US, the UK, Belgium, Israel, and Thailand.

Taken together, prior cross-national findings provide mixed evidence regarding the magnitude and expression of self-objectification and its correlates across countries, with some studies reporting higher levels in Western contexts (e.g., Loughnan et al., 2015), whereas others report more complex patterns (e.g., Crawford et al., 2009). Importantly, however, the limited number of existing cross-national studies is not well suited to drawing strong conclusions about how culture shapes objectification-related processes, as many rely on broad and simplified West–non-West distinctions. In line with recent calls to strengthen cumulative science in psychology by emphasizing the generalizability of theoretical models across contexts rather than culture-essentialist interpretations (Eronen & Bringmann, 2021), the present research adopts a generalizability perspective. Specifically, our primary objective is to test whether a key element of Objectification Theory (i.e., the mediating role of body surveillance in the association between self-objectification and body shame) holds across multiple national contexts. To this end, we examine this mediation model in samples from the United States, the United Kingdom, Belgium, Israel, and Thailand, thereby assessing the robustness of the proposed psychological process across both US and non-US settings, without assuming that observed cross-national differences necessarily reflect uniform or universal cultural mechanisms.

## Self-Objectification in the USA, UK, Belgium, Israel, and Thailand

Beauty standards in the UK, Belgium and Israel are heavily influenced by American beauty ideals (Talmon & Ginzburg, 2016; Wollast et al., 2018). Such standards have never been more pervasive than they are today (Karsay et al., 2017). US mass media play a major role in shaping women’s perception of their bodies by promoting an unattainable beauty that does not leave room for cultural and individual differences. In this context, research in Belgium has found that body surveillance is related to exposure to sexually objectifying media (e.g., music television, social networking sites, fashion magazines) among Belgian teenage girls (Vandenbosch & Eggermont, 2012). Similarly, Wollast et al. (2019, 2020a) found that body surveillance is positively associated with body shame among Belgian women. Similar patterns were found among UK and Israeli women (Talmon & Ginzburg, 2016, 2018).

Although Thailand was never formally colonized, influences from the West and from Indian thought systems on aesthetic ideals dominate East Asian beauty standards such as large eyes, pointy nose, thinness, and white skin (Chaipraditkul, 2013). For instance, the ideal of whiteness is widespread in Thailand as well as in other Asian countries. Specifically, women with fairer skin are more likely to marry partners of a higher social class, since white skin is associated with youthfulness, high-quality women, health, and wealth in Asia (Li et al., 2008). Specifically, Thai stereotypes are used to emphasize feminine beauty such that women gain respect through acts of sacrifice and beauty, which they possesses to glorify their husband (Chaipraditkul, 2013). Consequently, Thai women are taught from a young age to protect their virtue and innocence through their appearance, which may lead them to engage in self-objectification. Additionally, it is widely recognized that in numerous Asian societies, experiencing public loss of face is deemed unacceptable and can result in shame. This phenomenon happens when an individual, whether through their actions or those of closely associated individuals, fails to fulfill fundamental expectations associated with their social role (Ho, 1976). Given that appearance is an important factor in social ranking (Gervais et al., 2015), individuals may attempt to protect their reputation by comparing their physical appearance to that of others (i.e., body surveillance), circumventing negative feelings brought about by losing face (Chaipraditkul, 2013).

Taken together, these national differences in body image suggest that self-objectification is a widespread phenomenon that may manifest differently across national contexts, with some societies, such as the United States, placing a particularly strong emphasis on sexualized forms of appearance.

## The Present Research

In their literature review on self-objectification, Moradi and Huang (2008, p. 392) wrote that “most objectification theory studies are with samples from the United States and Australia. Research is needed to examine cultures in which women’s bodies are treated with more, less, and different manifestations of objectification.” Based on these recommendations, Study 1 adds to the literature on national differences by comparing the relations among self-objectification, body surveillance, and body shame among Belgian, UK, and US women using the validated Likert version of the Self-Objectification Questionnaire (Wollast et al., 2021). In Study 2, we aim at replicating the results of Study 1 in Belgium, Israel, and Thailand, where these associations remain understudied.

## Study 1

This research adheres to the ethical guidelines set by the American Psychological Association for conducting research involving human subjects. All participants provided informed consent online, following approval by the institutional review boards overseeing the principal investigators. The questionnaire and original data are accessible in the supplementary materials available online (https://osf.io/rjcea).

### Participants

In total, 611 female undergraduate students participated in this study. Of them, 239 were Belgian, 213 were British, and 159 were American. Participants’ ages ranged from 18 to 69 years (Belgian: *M* = 24.43, *SD* = 9.80; UK: *M* = 22.31, *SD* = 8.84; American: *M* = 19.21, *SD* = 1.07). In each country, participants were predominantly students, and there was little variation in their reported relationship/marital status (Belgian: 54% single, 37% in a committed relationship but not married, 6% married, 2% divorced, 1% widowed; UK: 54% single, 41% in a committed relationship but not married, 3% married, 1% divorced, 1% widowed; US: 81% single, 19% in a committed relationship but not married). Participants were recruited on their university campus, and the measures were completed in their respective native language. The participants completed the questionnaire online in exchange for course credits.

### Sample Size Determination and Power

Sample sizes ranged from *N* = 159–239 in Study 1 and *N* = 209–299 in Study 2, reflecting the size of the available participant pools within each recruitment context. We conducted a sensitivity analysis to assess statistical power. Using the smallest group size in the multi-group models (*N* = 159), the design provides approximately 80% power (two-tailed α = .05) to detect effects around r ≈ .22 (or equivalently, standardized paths of roughly β ≈ .22). Larger groups have correspondingly higher sensitivity. Because power for mediation depends on the magnitude of the component paths, indirect effects were evaluated using bootstrapped confidence intervals.

### Procedure

All instruments administered were validated (McKinley & Hyde, 1996; Wollast et al., 2021). The questionnaire included variables measuring the Likert version of self-objectification, body surveillance, and body shame. At the end of the questionnaire, participants were required to answer socio-demographic questions. After completing the questionnaire, participants were given the opportunity to request a full debriefing.

### Material

**LSOQ.** To assess self-objectification, we relied on the Likert version of the Self-Objectification Questionnaire (LSOQ, Wollast et al., 2021), which constitutes a psychometrically improved adaptation of the original Self-Objectification Questionnaire (SOQ; Noll & Fredrickson, 1998; Fredrickson et al., 1998). In the original SOQ, participants are asked to rank bodily attributes reflecting physical appearance versus physical competence according to their importance for the physical self-concept, thereby forcing a relative prioritization between these two dimensions (see exact instruction in Study 2 below). In contrast, the LSOQ preserves the same set of bodily attributes and the same theoretical definition of self-objectification, but replaces the ranking task with independent Likert-type ratings, allowing participants to evaluate the importance of each attribute without an imposed trade-off. Specifically, participants were instructed as follows: “The questions below identify 10 different bodily attributes. We would like you to indicate the extent to which each of these bodily attributes has an impact on your physical self-concept. For each attribute, please indicate whether the attribute has an extremely low or extremely high impact on your physical self-concept.” Participants rated each attribute on a visual analog scale ranging from 1 (*Low impact*) to 11 (*High impact*). Consistent with prior work, a self-objectification score was computed by subtracting the mean competence score from the mean appearance score, with higher values indicating greater self-objectification.^1^

**Body Surveillance and Body Shame.** We used two dimensions of the Body Objectified Consciousness Scale (McKinley & Hyde, 1996), and asked participants to complete the Body Surveillance subscale (e.g., “During the day, I think about how I look many times.”) and the Body Shame subscale (e.g., “When I can’t control my weight, I feel like something must be wrong with me.”). Participants rated eight items for each scale from 1 (*Strongly disagree*) to 7 (*Strongly agree*), with a higher averaged score indicating more body surveillance or body shame.

### Multi-group Measurement Invariance

Prior to comparing responses across groups, it was necessary to test whether Belgian, UK, and US participants interpreted the survey questions in a similar manner. To do so, we tested measurement invariance for the measurement model of body surveillance and body shame (with self-objectification included as an observed predictor in the structural model, Byrne, 2004). The adequacy of the model fit was evaluated using several fit indices, including the comparative fit index (CFI) and root mean square error of approximation (RMSEA), with thresholds set at CFI > .90 and RMSEA < .08 (Hu & Bentler, 1999). Given the comparison of three groups, changes in CFI and RMSEA of < .015 between models are considered indications of invariance (Chen, 2007; Cheung & Rensvold, 2002).

First, an unconstrained configural model was employed to verify that the general factor structure of the measure is the same for Belgian, UK, and US by freely estimating parameters in each of the groups. This unconstrained model constitutes a basis for comparison to test for invariance. Then, a constrained metric model was run in which measurement weights of the latent variables (i.e., factor loadings) were constrained to be equal. Finally, an additional constrained scalar model was run in which the factor loadings and intercepts were constrained to be equal. To determine whether measurement invariance is present, or whether the same construct is being measured across groups, we tested metric invariance by comparing the metric model with constrained measurement weights to the unconstrained configural model and tested scalar invariance by comparing the scalar model with constrained intercepts to the metric model with constrained measurement weights (Byrne, 2004). If scalar invariance is not achieved, a partial intercepts invariance model can be estimated, in which the intercept of an item is estimated freely across groups. The intercepts have to be invariant for at least two items per variable (Coertjens et al., 2012).

Table 1 reports the fit indices for the tested measurement invariance models. Measurement invariance analyses for the full model were successfully conducted and supported partial scalar invariance, indicating that the measurement models were largely comparable across Belgian, UK, and US women, thereby allowing meaningful cross-group comparisons of latent means. Importantly, composite reliability ranged from acceptable to good for body surveillance (ω_Belgian_ = .77; ω_UK_ = .86; ω_US_ = .87), was good for body shame (ω_Belgian_ =.87; ω_UK_ = .87; ω_US_ = .88) as well as for the appearance (ω_Belgian_ = .69; ω_UK_ = .76; ω_US_ = .74) and competence (ω_Belgian_ = .69; ω_UK_ = .76; ω_US_ = .74) dimensions of the LSOQ.

**Table 1:** Fit indices for multi-group measurement invariance models across studies.


TESTED MODELS	CHARACTERISTICS	χ^2^	DF	CFI	RMSEA

*Study 1*					

Configural	Unconstrained model	544.287	285	.937	.039

Metric	Equal factor loadings	641.581	313	.918	.042

Scalar	Equal intercepts	944.296	345	.854	.053

Partial scalar	Partial equal intercepts	720.014	321	.903	.045

*Study 2*					

Configural	Unconstrained model	268.609	156	.959	.031

Metric	Equal factor loadings	336.629	178	.942	.035

Scalar	Equal intercepts	868.159	204	.758	.067

Partial scalar	Partial equal intercepts	403.856	186	.920	.040


### Results

Descriptive statistics and zero-order correlations based on manifest scores are presented in Table 2 for completeness, whereas all inferential analyses reported below rely on latent variable modeling.

**Table 2:** Descriptive statistics and zero-order correlations for self-objectification, body surveillance, and body shame across studies and countries.


		BODY SURVEILLANCE	BODY SHAME	MEAN (SD)

Belgium(Study 1)	LSOQ	.39**	.21**	–0.48 (1.72)

	Body Surveillance	–	.39**	4.77 (1.05)

	Body Shame	–	–	3.00 (1.42)

UK(Study 1)	LSOQ	.52**	.41**	.50 (1.94)

	Body Surveillance	–	.59**	5.14 (1.11)

	Body Shame	–	–	4.32 (1.29)

US(Study 1)	LSOQ	.63**	.44**	0.17 (2.21)

	Body Surveillance	–	.46**	4.96 (1.17)

	Body Shame	–	–	3.61 (1.17)

Belgium(Study 2)	SOQ	.50**	.30**	1.38 (13.71)

	Body Surveillance	–	.53**	4.77 (1.02)

	Body Shame	–	–	3.09 (1.15)

Israel(Study 2)	SOQ	.15**	.08	–1.89 (8.82)

	Body Surveillance	–	.46**	4.83 (1.07)

	Body Shame	–	–	3.22 (1.21)

Thailand(Study 2)	SOQ	.31**	.19**	–5.41 (10.86)

	Body Surveillance	–	.24**	4.00 (0.98)

	Body Shame	–	–	3.44 (0.86)


Note: **p < .01. LSOQ = Likert Version of the Self-Objectification Questionnaire. SOQ = the Self-Objectification Questionnaire. For descriptive purposes, means and correlations are reported using manifest scores computed from the full original item sets of each scale. Inferential analyses were conducted using latent variables as specified in the SEM models.

We tested whether the latent means differed significantly among Belgian, UK, and US women. To do so, latent mean invariance was conducted under strict invariance, with factor loadings, intercepts, residuals, and latent factor variances constrained to be equal across groups.

When constraining the means to zero for UK women, the results indicated that US women scored significantly lower on the body shame scale (Estimate = –.595, *SE* = .144, *p* < .001), and Belgian women scored even lower (Estimate = –1.329, *SE* = .145, *p* < .001). It is noteworthy that Belgians also scored significantly lower than US women (Estimate = –.881, *SE* = .168, *p* < .001).

Interestingly, no differences were observed for the body surveillance scale between UK and US women (Estimate = .115, *SE* = .087, *p* = .185) or Belgian women (Estimate = .139, *SE* = .098, *p* = .158). Similarly, US and Belgian women did not differ in their levels of body surveillance (Estimate = .050, *SE* = .098, *p* = .611).

Regarding the LSOQ, using post-hoc Tukey’s tests with Games-Howell correction, we found that the groups differed significantly (*F*(2, 608) = 14.913, *p* < .001, *η_p_*^2^ = .047). Specifically, UK (*M* = .50, *SE* = 1.94) and US (*M* = .17, *SE* = 2.21) women scored significantly higher than Belgian women (*M* = -.48, *SD* = 1.73), but they did not differ between each other.

When looking at the mean scores of the LSOQ items, it became apparent that women from the three countries attributed greater importance to physical attractiveness, weight, sex appeal, and health compared to the other attributes. Specifically, in the UK sample, the top five mean scores were as follows: Physical attractiveness (*M* = 9.20), Weight (*M* = 8.98), Sex appeal (*M* = 7.74), Health (*M* = 8.30), Physical fitness (*M* = 8.05). In the US sample, the mean scores were as follows: Physical attractiveness (*M* = 9.52), Weight (*M* = 8.46), Sex appeal (*M* = 8.04), Health (*M* = 8.68), Physical fitness (*M* = 8.16). For the Belgian sample, we observed: Health (*M* = 9.41), Physical attractiveness (*M* = 8.70), Physical fitness (*M* = 7.81), Weight (*M* = 7.53), Energy level (*M* = 7.35).

**Figure 1:**
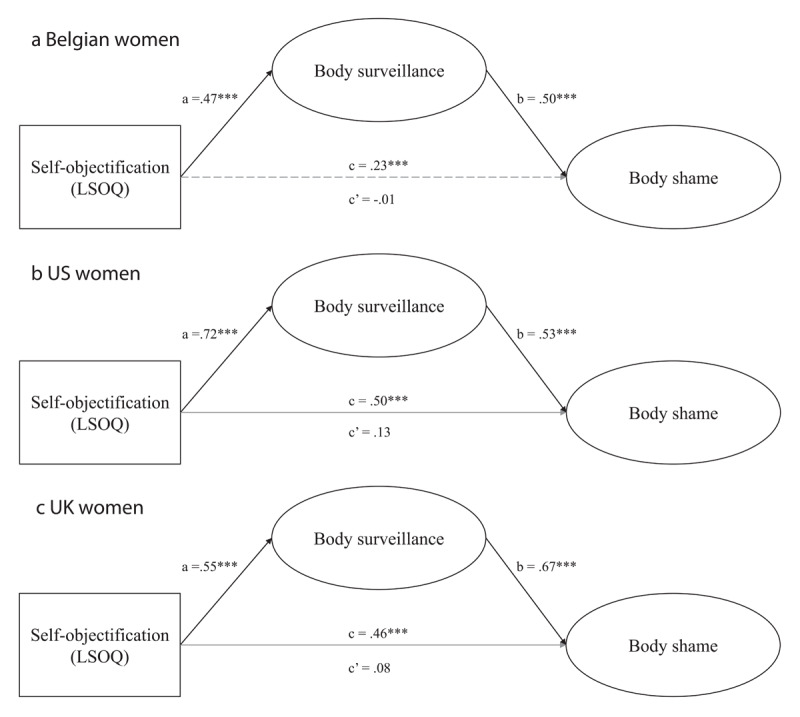
Standardized regression coefficients for the relationship between self-objectification and body shame as mediated by body surveillance among Belgian **(a)**, US **(b)**, and UK **(c)** women (Study 1).

**Hypothesized Structural Equation Model.** Subsequently, a multiple group structural equation model with country as a factor was estimated to test for the hypothesized mediation. Although the parameter estimates for the Belgian, UK, and US women stem from one SEM model, for clarity, we opted to present them in Figure 1. The SEM model provided good model fit statistics (*χ*^2^ = 544.287, *df* = 285, *CFI* = .937, *RMSEA* = .039).

In Step 1 of the mediation model, the regression of self-objectification on body shame, ignoring the mediator (i.e., total effect), was significant for all Belgian, UK, and US women (see path c on the figures). Step 2 showed that the regression of self-objectification on the mediator, body surveillance, was significant for each group (see path a on the figures). Similarly, Step 3 of the mediation process showed that the mediator (body surveillance), controlling for self-objectification, was significant for each group (see path b on the figures). As expected, Step 4 of the analyses revealed that controlling for the mediator (body surveillance), the effect of self-objectification on body shame becomes non-significant for each group (see path c’ on the figures) and strongly decreases (*ΔB* = .23*** for Belgians, *ΔB* = .38*** for UK women, and *ΔB* = .37*** for US women). Further, an analysis of indirect effects demonstrated that self-objectification indirectly predicted body shame via body surveillance for Belgians (*B* = .401, *SE* = .096, *p* = .008, 95% CI [.241; .608]), UK women (*B* = .426, *SE* = .097, *p* = .016, 95% CI [.232; .606]), and US women (*B* = .603, *SE* = .189, *p* = .018, 95% CI [.155; .913]). In summary, confirming our hypotheses, we found that body surveillance mediated the relationship between self-objectification and body shame among Belgian, UK, and US women.

## Study 2

Although Wollast et al. (2021) raised concerns about the psychometric limitations of the original ranking format of the Self-Objectification Questionnaire (SOQ; see also Calogero, 2011), the SOQ remains widely used in the literature. Accordingly, Study 2 further tests the generalizability of the hypothesized mediation model across additional national contexts beyond the United States by examining Belgian, Israeli, and Thai women, while assessing self-objectification with the original SOQ ranking procedure (Fredrickson et al., 1998).

### Participants

In total, 738 female undergraduate students participated in this study. Of them, 209 were Belgian, 299 were Israeli, and 230 were Thai. Participants’ ages ranged from 18 to 60 years (Belgium: *M* = 21.25 *SD* = 5.41; Israel: *M* = 25.03 *SD* = 4.69; Thailand: *M* = 24.37 *SD* = 6.80). In each country, participants were predominantly students, and there was little variation in their reported marital status (Belgium: 61% single, 37% in a committed relationship but not married, 2% married; Israel: 66% single, 32% in a relationship or married; 2% divorced; Thailand: 85% single, 7% in a committed relationship but not married, 6% married, 2% widowed). Participants were recruited on their university campus, and the measures were completed in their respective native language. We also posted the online survey on students’ corresponding university work groups to collect more participants. The participants completed the questionnaire online on a voluntary basis.

### Procedure

Instruments used in the Belgian, Israeli, and Thai samples were subjected to back-translation by independent translators who were uninformed about the objectives of the current study. This methodical translation approach was chosen to ensure that the constructs, rather than exact wording, maintained equivalence across languages. The questionnaire included items assessing self-objectification, body surveillance, and body shame. Participants were also asked to provide socio-demographic information at the end of the questionnaire. Following completion, participants had the option to request a comprehensive debriefing.

### Measures

We used the Body Surveillance and Body Shame scales from Study 1.

**Self-Objectification.** We used the Self-Objectification Questionnaire (SOQ; Fredrickson et al., 1998). Participants assessed the significance of five visible appearance-related attributes (physical attractiveness, weight, sex appeal, measurements, firm/sculpted muscles) compared to five non-visible competence-related attributes (health, strength, energy level, physical coordination, physical fitness). In line with Fredrickson et al.’s approach, the difference between these ranks was used as an indicator of self-objectification. If participants ranked observable attributes as more important, the non-observable attributes would be ranked as less important (i.e., 9 if the most important is visible or 0 if the most important is non-observable, 8 for the second-most important, and so on). The scores range from –25 to +25, with a higher score indicating more self-objectification. The variable was standardised prior to inclusion into the SEM model.

### Multi-group Measurement Invariance

As in Study 1, prior to comparing responses across groups, it was necessary to test whether Belgian, Israeli, and Thai participants interpreted the survey questions in a similar manner. To do so, we estimated different levels of measurement invariance for a model including self-objectification, body surveillance, and body shame (Byrne, 2004).

Table 1 reports fit indices for each model (see bottom panel for Study 2). First, we tested measurement invariance using an unconstrained model including all items and correlated errors, in which three items of body shame that loaded too differently in the Thai sample were excluded (see retained items in the online supplementary material). This configural model revealed good model fit statistics (see Table 1). Second, we compared this configural model with a constrained model which confirmed that metric invariance was achieved. Third, we created an additional constrained scalar model with constrained intercepts and loadings, which led to a decrease in model fit statistics. Partial intercept invariance models were estimated, and, after freely estimating nine intercepts, model fit was restored approximately to the level of the metric invariance model. Ultimately, internal consistency ranged from acceptable to good for body surveillance (ω_Belgian_ = .77; ω_Israeli_ = .84; ω_Thai_ = .78) and ranged from low to good for body shame (ω_Belgian_ = .81; ω_Israeli_ = .83; ω_Thai_ = .57).

### Results

Descriptive statistics and zero-order correlations based on manifest scores are presented in Table 2 for completeness, whereas all inferential analyses reported below rely on latent variable modeling.

**Latent Means Differences.** We tested whether the latent means differed significantly among Belgian, Israeli, and Thai women. To do so, latent mean invariance was conducted under strict invariance, with factor loadings, intercepts, residuals, and latent factor variances constrained to be equal across groups. Unless otherwise indicated, the means were constrained to zero for Thai women.

First, results indicated that Thai women scored significantly lower on the body surveillance scale than Belgian (Estimate = .656, *SE* = .086, *p* < .001) and Israeli (Estimate = .782, *SE* = .085, *p* < .001) women, whereas the latter two did not differ significantly (Estimate = .145, *SE* = .078, *p* = .127, when the mean was constrained to zero for Belgians).

Second, we found that Thai women scored significantly higher on the body shame scale than Belgian (Estimate = –1.055, *SE* = .128, *p* < .001) and Israeli (Estimate = –1.246, *SE* = .111, *p* < .001) women, whereas the latter two did not differ significantly (Estimate = –.141, *SE* = .077, *p* = .172, when the mean is constrained to zero for Belgians). Thus, although Thai women reported lower levels of body surveillance and self-objectification, they nonetheless reported higher levels of body shame.

Third, given the ranking format of the SOQ, this variable was not estimated as a latent variable but rather as a manifest variable. We relied on Welch’s F-test when comparing our independent groups. We found a main effect of country on self-objectification (*F*[2, 462.432] = 16.63, *p* < .001, *η_p_*^2^ = .05). For comparing group means, we utilized post-hoc comparisons employing Tukey’s HSD with Games-Howell’s adjustment. Post-hoc tests revealed that Belgian participants (*M* = 1.38, *SD* = 13.71) obtained the highest SOQ scores, followed by Israelis (*M* = –1.89, *SD* = 8.82) and then Thais (*M* = –5.41, *SD* = 10.86).

An inspection of the SOQ items’ frequencies among Belgian, Israeli, and Thai women revealed similarities and differences in the order of ranked items (see Table S1). Regarding the observable attributes, Belgian and Israeli women valued their physical attractiveness and weight more than Thai women, while Thai women still considered physical attractiveness but also measurements as their most important observable attributes. As for non-observable attributes, the pattern is consistent: Health appeared to hold the most importance in each group. While physical coordination is considered important in the three countries, Thai women ranked this attribute higher than Belgian and Israeli women.

**Figure 2:**
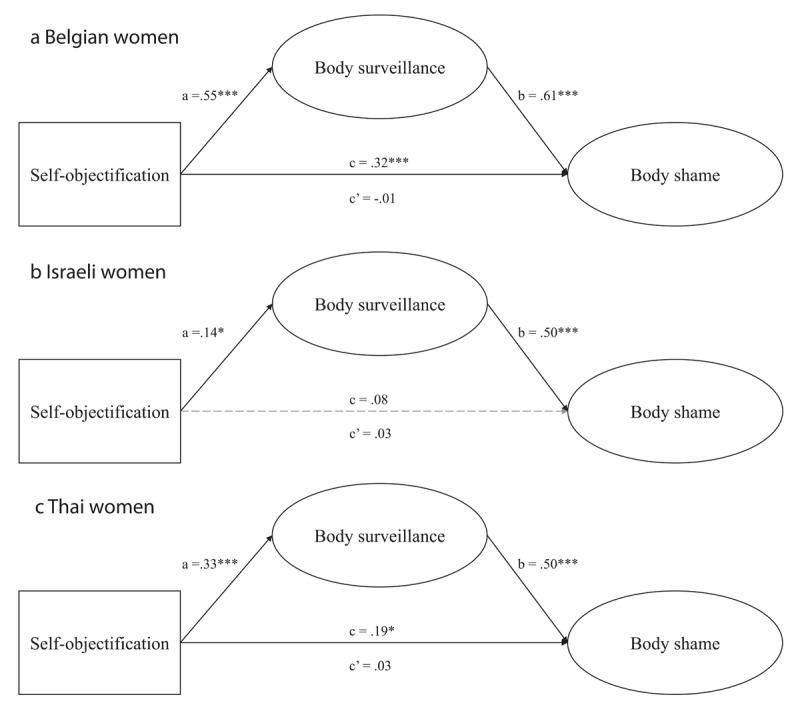
Standardized regression coefficients for the relationship between self-objectification and body shame as mediated by body surveillance among Belgian **(a)**, Israeli **(b)**, and Thai **(c)** women (Study 2).

**Hypothesized Structural Equation Model.** Replicating the same analysis strategy as in Study 1, a multiple group structural equation model with country as a factor was estimated to test for the hypothesized mediation. Although the parameter estimates for the Belgian, Israeli and Thai participants stem from one SEM model, for clarity, we opted to present them in three figures (see Figure 2). The SEM model provided excellent fit in terms of the RMSEA (.039; 90% CI = .034–044) and adequate fit in terms of the CFI (.923).

In Step 1 of the mediation model, the regression of self-objectification on body shame, ignoring the mediator (i.e., total effect), was significant for both Belgian and Thai women, but not for Israeli women (see path c on the figures). Step 2 showed that the regression of self-objectification on the mediator, body surveillance, was significant for each group (see path a on the figures). Similarly, Step 3 of the mediation process showed that the mediator (body surveillance), controlling for self-objectification, was significant for each group (see path b on the figures). Step 4 of the analyses revealed that, controlling for the mediator (body surveillance), self-objectification was not a significant predictor of body shame for each group (see path c’ on the figures). Further, an analysis of indirect effects demonstrated that self-objectification indirectly predicted body shame via body surveillance for Belgians (*B* = .386, *SE* = .122, *p* = .003, 95% CI [.220; .744]), Israelis (*B* = .079, *SE* = .043, *p* = .046, 95% CI [.005; .183]), and Thais (*B* = .096, *SE* = .036, *p* = .001, 95% CI [.046; .159]).

In sum, it was found that body surveillance fully mediated the relationship between self-objectification and body shame for Belgian and Thai women. This relationship could not be established for Israeli women but an indirect effect through body surveillance was found. This discrepancy may be explained by unobserved mediating (or suppressor) variables that operate on the total effect (see Hayes, 2009). Altogether, our hypothesized model, suggesting that the impact of self-objectification on body shame is explained in part by body surveillance, finds cross-national support in the present data.

## General Discussion

Research examining self-objectification is flourishing. However, little is known about self-objectification and its manifestations from a cross-national perspective. Thus, we looked beyond the typical objectification participant sample and surveyed women from the United States of America, the United Kingdom, Belgium, Israel, and Thailand. Overall, the results indicated that self-objectification, body surveillance, and body shame are strongly associated across countries. Notably, in Study 1, employing the Likert version of the Self-Objectification Questionnaire (LSOQ), we demonstrated that self-objectification indirectly predicts body shame through body surveillance in the case of Belgian, UK, and US women. In Study 2, we successfully replicated these indirect effects among Belgian, Israeli, and Thai women.

### Self-Objectification, Body Surveillance, and Body Shame

We found different levels of body surveillance and body shame across countries. Overall, women in the United Kingdom and the United States tended to report relatively higher levels of body surveillance and body shame compared to women from the other countries. In contrast, Belgian and Israeli women displayed comparatively higher body surveillance scores than Thai women, while simultaneously reporting lower levels of body shame. Notably, Belgian women consistently reported particularly low body shame across both studies, despite moderate to high levels of body surveillance. This pattern replicates earlier findings by Wollast et al. (2020b), who showed that American, Belgian, and Russian women reported higher body surveillance than Thai women, whereas Belgian women demonstrated the lowest levels of body shame. These discrepancies may reflect cross-national differences in how body-related self-conscious emotions are experienced and labeled. In line with this interpretation, qualitative comments provided by Belgian participants suggested that although they experienced discomfort or dissatisfaction with their bodies, they were reluctant to describe these experiences in terms of “shame,” which was perceived as excessively strong or inappropriate. A similar dissociation between body surveillance and body shame has been documented by Crawford et al. (2009), who found that Nepalese women engaged in less body surveillance but reported higher body shame than women from the United States. While U.S. women’s higher body surveillance was attributed to greater exposure to sexually objectifying media, anthropological accounts suggest that body shame among Nepalese women may be more closely tied to religious and cultural norms surrounding the female body, particularly in relation to purity, childbirth, and menstruation (Crawford et al., 2009). Taken together, these findings indicate that body surveillance and body shame are salient across national contexts but do not necessarily covary in the same manner. Instead, their expression appears to be shaped by the social meanings and functions attached to appearance, self-evaluation, and morality within each cultural context.

From a broader cultural perspective, anthropological and cultural psychological traditions have long distinguished between shame- and guilt-oriented cultures (Benedict, 1946; Markus & Kitayama, 1991). In many Asian contexts, shame functions as a central, socially regulated emotion tied to moral evaluation, social harmony, and the preservation of face (Wong & Tsai, 2007). From this perspective, body shame may represent only one specific manifestation of a more general cultural orientation toward shame, rather than being solely rooted in processes of sexual objectification. This may help explain why Thai women reported relatively high levels of body shame despite lower levels of self-objectification and body surveillance, suggesting that cultural norms surrounding shame extend beyond appearance-based self-evaluation.

Furthermore, patterns of self-objectification closely mirrored those observed for body surveillance across countries. In Study 1, we found strong similarities as UK and US women obtained higher scores than Belgians. In Study 2, we observed that Belgians and Israelis manifest higher levels of self-objectification than Thais. While they all value their observable attributes (i.e., the body appearance dimension), self-objectification tended to be more pronounced in Western or Western-oriented contexts, particularly in the United States and the United Kingdom. At the attribute level, physical attractiveness and weight were valued more strongly by women from Western countries than by Thai women, whereas Thai women attributed relatively greater importance to measurements. Regarding non-observable attributes (i.e., the body competence dimension), all women considered health to be their most important attribute (for a discussion of the centrality of health in self-objectification measures, see Wollast et al., 2021). This general pattern aligns with findings by Loughnan et al. (2015), who reported higher levels of self-objectification in Western compared to non-Western contexts.

Importantly, these cross-national differences may also reflect limitations of the measurement instrument itself. The SOQ (Fredrikson et al., 1998) was designed to study self-objectification in Western countries and especially in American culture. It refers to observable attributes (e.g., physical attractiveness, weight, sex appeal) that directly relate to Western beauty standards. Other appearance dimensions may be more salient in non-Western contexts. For instance, skin tone plays a central role in beauty standards across many Asian societies, where lighter skin is associated with status, health, and femininity (Li et al., 2008). Because skin tone is not included among the SOQ attributes, the measure may insufficiently capture culturally specific manifestations of self-objectification among Thai women (see also Buchanan et al., 2008).

Moreover, while Belgium and Israel are strongly influenced by Western beauty norms (e.g., thinness), aesthetic ideals in Thailand reflect a hybrid of Western, Indian, and East Asian influences (e.g., emphasis on facial features such as a pointed nose). Consequently, lower SOQ scores among Thai women may not indicate lower self-objectification, but rather reduced alignment between culturally salient appearance ideals and the attributes assessed by the SOQ.

### The Mediating Role of Body Surveillance

The present research is also highlighting a robust a mediating role of body surveillance in the relation between self-objectification and body shame in Belgium and Thailand, and successfully replicate this pattern among US and UK women. These findings strengthen objectification theory (Fredrickson & Roberts, 1997) and objectified body consciousness (McKinley & Hyde, 1996) in understudied national contexts. While this mediation effect did not emerge in the Israeli sample, the indirect effect was significant for all women across the five countries, providing evidence that self-objectification indirectly predicts body shame through body surveillance, across national backgrounds. The occurrence of these indirect effects across countries may be explained by the high levels of sexism, gender inequalities, and endorsement of traditional gender roles in the investigated countries (Glick et al., 2000).

Specifically, Calogero and Jost (2011) demonstrated that broad cultural ideologies justifying gender inequality and preserving the gender status quo trigger self-objectification. More precisely, self-objectification might be activated by broader environmental antecedents that convey information about culturally prescribed gender roles and behaviors (Calogero & Jost, 2011). Indeed, a primary aspect of the feminine gender role is to look attractive (Mahalik et al., 2005), consequently leading women to self-objectify. This suggests that women who endorse such traditional gender roles value their appearance more and are more likely to engage in self-objectification and surveillance of their own bodies. According to Chaipraditkul (2013, p. 2), “In most of Asia, the fairer skin one has, the higher the future marriage partner’s social class could be. Fair skin is believed to symbolize youthfulness and high quality in women. In Thailand, fair skin symbolizes health and wealth.” In sum, beauty is extremely important for Thai women because it can lead to social gratification and power (Chaipraditkul, 2013). A similar pattern occurs in the US, UK, Belgium and Israel, in which physical attractiveness can also be used by women as a means for attaining social power (De Wilde et al., 2020; Liss et al., 2010). Furthermore, in patriarchal societies that subordinate women, those who do not conform to such beauty ideals may experience detrimental outcomes (e.g., discrimination, fat shaming), whereas women who have an appearance that is consistent with gender norms are rewarded (e.g., Sibley & Wilson, 2004). In this respect, self-objectification and body surveillance, followed by body shame, can occur as an internalized alarm to remind women that they must conform to traditional gender norms prescribed in their societies. In this context, we encourage researchers to rely on greater panels of countries to investigate the relationship between country-level indicators of gender equality (e.g., Gender Inequality Index, Gender Development Index), perceived gender inequalities, and self-objectification.

### Limitations and Future Directions

The findings of the present study should be interpreted while considering some limitations. First, the survey includes mostly undergraduate students (90%) in each country. Although this is common practice throughout psychology, it limits the external validity of the results (Henrich et al., 2010).

Second, although overall reliability was acceptable, McDonald’s omega for body shame was lower in the Thai sample. Measurement invariance analyses indicated that three of the eight body shame items did not function equivalently and were therefore excluded. Importantly, this non-invariance is more likely to reflect linguistic and measurement issues than substantive cultural differences. Two of the excluded items involved negatively worded or reverse-coded formulations and abstract moral self-evaluations (e.g., “I never worry that something is wrong with me when I am not exercising as much as I should.”), which are known to be particularly difficult to translate and interpret consistently across languages (Swain et al., 2008). These items were already challenging during the translation process and likely contributed to their weaker psychometric performance. In contrast, the retained items captured a more direct, judgment-based dimension of body shame that generalized more robustly across groups (e.g., “I feel ashamed of myself when I do not make an effort to look my best.”). This highlights the importance of multi-group measurement invariance testing in cross-national research and highlights the need for careful cultural and linguistic validation of psychological measures.

Third, self-objectification could not be examined in terms of cross-national measurement invariance because both the original Self-Objectification Questionnaire (SOQ) and its Likert adaptation (LSOQ) were modeled as manifest variables in the present analyses, yielding a single composite score of self-objectification. This modeling choice precludes testing whether the measurement structure of self-objectification is equivalent across national groups. Importantly, self-objectification can also be conceptualized as a bidimensional construct, comprising separable appearance-based and competence-based dimensions (Wollast et al., 2021). Future research could therefore model these two dimensions as distinct latent variables within the same structural equation model, which would allow for formal tests of measurement invariance across cultures. Although such an approach would substantially increase model complexity, it would provide a more precise assessment of self-objectification across cultural contexts.

Fourth, given the cross-sectional nature of the present studies, causal inferences cannot be drawn. It is also possible that the relationships between self-objectification, body surveillance, and body shame are bidirectional, such that heightened body shame may in turn reinforce increased appearance monitoring and related processes (see Kilpela et al., 2019). Longitudinal and experimental designs (e.g., group-based trajectory modeling, see Wollast et al., 2025) will be necessary to disentangle these dynamic and potentially reciprocal associations.

This study has important implications for negative body image and health-related behaviors among women from different national backgrounds. It suggests that women’s body image manifests itself differently across countries. This raises the question: Which aspects of a national background will influence the way people see themselves? Yet, the variables that might explain the national differences in self-objectification have not yet been the focus of extensive research in the current cross-national literature. In line with the role of sexism in self-objectification described above, another key factor that may shape body surveillance and body shame across countries is media exposure (Karsay et al., 2017; Di Michele et al., 2023). It is well known that Western (Ward, 2016) and Eastern (Wollast et al., 2018) cultures set unrealistic appearance ideals in the media. For instance, the export of Korean TV dramas across Asian countries, including Thailand, has a considerable impact on the formation of gender, femininity, beauty norms, and class identities (Yang, 2008). Moreover, it has been established that exposure to idealized, stereotyped, or sexualized images socializes individuals to take an outsider’s perspective on their physical selves and self-objectify, potentially leading to detrimental consequences (e.g., Krawczyk & Thompson, 2015, see also Bernard et al., 2020). An investigation of the association between exposure to objectifying media and self-objectification, in a cross-national framework, may provide greater perspectives on how self-objectification manifest cross-nationally.

Finally, there is a growing body of research using the Likert version of the Self-Objectification Questionnaire (LSOQ), which preserves the face validity of the original SOQ while offering improved psychometric properties. Since its validation, the LSOQ has already been adopted in several empirical studies (e.g., Bachner-Melman et al., 2024; Cervone et al., 2025; Kranz, 2025), supporting its usefulness across diverse research contexts. We encourage scholars to continue advancing research in this direction. At the same time, our findings align with recent work highlighting the limitations of the original Self-Objectification Questionnaire (SOQ; Fredrickson et al., 1998) and support the use of alternative instruments with improved psychometric properties, such as the Self-Objectification Beliefs and Behaviors Scale (Lindner & Tantleff-Dunn, 2017), which has similarly demonstrated advantages over the SOQ (see Kahalon et al., 2024).

## Conclusions

This paper offers multiple contributions to the understanding of the phenomenon of self-objectification. The purpose of this research was to examine cross-national comparisons of self-objectification, body surveillance, and body shame in the United States of America, United Kingdom, Belgium, Israel, and Thailand. Based on the analysis conveyed, it can be concluded that self-objectification and its related constructs are strongly associated within each national contexts but manifest themselves differently as a function of cultural variations. Additionally, the present work is one of the first empirical and cross-national investigations of body image concerns using the validated Likert version of the Self-Objectification Questionnaire (LSOQ, Wollast et al., 2021). The replicated indirect effects as well as the good model fit statistics suggest that the LSOQ is a reliable and valid measure of self-objectification. In this context, we strongly encourage scholars to systematically rely on this improved scale when they assess self-objectification. We hope this study can encourage researchers to adopt a cross-national perspective to bolster the current literature and enrich the debate in the field of objectification.

## Data Accessibility Statements

Data and supplementary material supporting this study are openly available from Open Science Framework (OSF) at https://osf.io/rjcea.

## Footnotes

It is worth mentioning that all results have been successfully replicated when only considering the body appearance variable (i.e., utilizing only 5 items from one dimension of the LSOQ) as independent variable. However, when considering a total self-objectification score incorporating both dimensions, the mediation effects were stronger.
